# Research on the Epidemiology of SARS-CoV-2 in Essential Response Personnel (RECOVER): Protocol for a Multisite Longitudinal Cohort Study

**DOI:** 10.2196/31574

**Published:** 2021-12-03

**Authors:** Laura J Edwards, Ashley L Fowlkes, Meredith G Wesley, Jennifer L Kuntz, Marilyn J Odean, Alberto J Caban-Martinez, Kayan Dunnigan, Andrew L Phillips, Lauren Grant, Meghan K Herring, Holly C Groom, Karley Respet, Shawn Beitel, Tnelda Zunie, Kurt T Hegmann, Archana Kumar, Gregory Joseph, Brandon Poe, Paola Louzado-Feliciano, Michael E Smith, Matthew S Thiese, Natasha Schaefer-Solle, Young M Yoo, Carlos A Silvera, Julie Mayo Lamberte, Josephine Mak, L Clifford McDonald, Matthew J Stuckey, Preeta Kutty, Melissa L Arvay, Sarang K Yoon, Harmony L Tyner, Jefferey L Burgess, Danielle Rentz Hunt, Jennifer Meece, Manjusha Gaglani, Allison L Naleway, Mark G Thompson

**Affiliations:** 1 Abt Associates, Inc Atlanta, GA United States; 2 Centers for Disease Control and Prevention Atlanta, GA United States; 3 Kaiser Permanente Northwest Center for Health Research Portland, OR United States; 4 Whiteside Institute for Clinical Research Duluth, MN United States; 5 St. Luke’s Regional Health Care System Duluth, MN United States; 6 University of Miami Miami, FL United States; 7 Baylor Scott and White Health Temple, TX United States; 8 University of Utah Salt Lake City, UT United States; 9 University of Arizona Tucson, AZ United States; 10 Marshfield Clinic Research Institute Marshfield, WI United States; 11 Texas A&M University College of Medicine Temple, TX United States

**Keywords:** COVID-19, SARS-CoV-2, incidence, vaccine effectiveness, cohort study, health care personnel, first responder, essential and frontline workers

## Abstract

**Background:**

Workers critical to emergency response and continuity of essential services during the COVID-19 pandemic are at a disproportionally high risk of SARS-CoV-2 infection. Prospective cohort studies are needed for enhancing the understanding of the incidence of symptomatic and asymptomatic SARS-CoV-2 infections, identifying risk factors, assessing clinical outcomes, and determining the effectiveness of vaccination.

**Objective:**

The Research on the Epidemiology of SARS-CoV-2 in Essential Response Personnel (RECOVER) prospective cohort study was designed to estimate the incidence of symptomatic and asymptomatic SARS-CoV-2 infections, examine the risk factors for infection and clinical spectrum of illness, and assess the effectiveness of vaccination among essential workers.

**Methods:**

The RECOVER multisite network was initiated in August 2020 and aims to enroll 3000 health care personnel (HCP), first responders, and other essential and frontline workers (EFWs) at 6 US locations. Data on participant demographics, medical history, and vaccination history are collected at baseline and throughout the study. Active surveillance for the symptoms of COVID-19–like illness (CLI), access of medical care, and symptom duration is performed by text messages, emails, and direct participant or medical record reports. Participants self-collect a mid-turbinate nasal swab weekly, regardless of symptoms, and 2 additional respiratory specimens at the onset of CLI. Blood is collected upon enrollment, every 3 months, approximately 28 days after a reverse transcription polymerase chain reaction (RT-PCR)–confirmed SARS-CoV-2 infection, and 14 to 28 days after a dose of any COVID-19 vaccine. From February 2021, household members of RT-PCR–confirmed participants are self-collecting mid-turbinate nasal swabs daily for 10 days.

**Results:**

The study observation period began in August 2020 and is expected to continue through spring 2022. There are 2623 actively enrolled RECOVER participants, including 280 participants who have been found to be positive for SARS-CoV-2 by RT-PCR. Enrollment is ongoing at 3 of the 6 study sites.

**Conclusions:**

Data collected through the cohort are expected to provide important public health information for essential workers at high risk for occupational exposure to SARS-CoV-2 and allow early evaluation of COVID-19 vaccine effectiveness.

**International Registered Report Identifier (IRRID):**

DERR1-10.2196/31574

## Introduction

SARS-CoV-2, the virus causing the COVID-19 global pandemic, has rapidly spread throughout the world and has led to over 3 million deaths since December 2019 [1,2]. Evidence from prospective cohort studies is critical for understanding the incidences of symptomatic and asymptomatic SARS-CoV-2 infections, identifying risk factors, assessing clinical outcomes, and determining the effectiveness of vaccination. Studies have suggested that the incidence of asymptomatic SARS-CoV-2 infection is high, but it is unclear whether or not individuals remain asymptomatic throughout their infection [3,4]. Additionally, while rare, reinfection has been documented among healthy adults with an indication of diminishing severity that may require systematic monitoring to identify asymptomatic infections [5]. Moreover, emerging variants of SARS-CoV-2 are causing concern and have been associated with an increase in the incidence of infection and may have implications for COVID-19 vaccine effectiveness [6-8]. Research is needed to improve our understanding of SARS-CoV-2 to better inform public health policies that may help protect essential workers and their patients, co-workers, customers, and household members, as well as others in the community, as they respond to the pandemic.

Essential workers, including health care personnel (HCP), first responders, and other essential and frontline workers (EFWs), serve in vital roles requiring direct interaction with the public to maintain the minimum requirements for a functional society. HCP include physicians, physicians’ assistants, nurse practitioners, dentists, medical assistants, medical technicians, nurses, nursing assistants, occupational therapists, pharmacists, and nonclinical personnel, including but not limited to social workers, receptionists, etc. First responders are workers in care or public contact response roles, including emergency medical technicians, firefighters, law enforcement, security guards, and other responders to emergency situations. EFWs are those not in HCP or first responder occupations, who perform work that cannot be executed from home and cannot be done without contact with other people. These include grocery store clerks, food service workers, those in certain agricultural and manufacturing occupations, hospitality employees, transportation and construction workers, and school teachers, among others. In a pandemic, these workers provide direct patient care, respond to the public need for assistance, and maintain critical public and private services. Work-related risks and exposures in essential workers differ from those in the general public, placing them at higher risk for exposure, infection, and transmission of SARS-CoV-2 [9-14]. The increased risk of infections, such as influenza, has previously been recognized for HCP and first responders, but few studies have been conducted to assess EFW infection risk or adherence and attitudes toward personal protective equipment (PPE), the use of which has been recommended as part of the COVID-19 pandemic response. A comprehensive assessment of these broad occupational categories is needed to understand infection risk, appropriate control measures, and COVID-19 vaccine effectiveness.

Vaccination against COVID-19 is an important tool for reducing both SARS-CoV-2 infections and the morbidity and mortality impacts associated with these infections. The Advisory Committee on Immunization Practices identified certain essential workers (particularly HCP and first responders) for earlier receipt of COVID-19 vaccines, making this population a high priority for studies evaluating vaccine effectiveness in preventing SARS-CoV-2 infection and transmission [15].

We designed a prospective cohort study entitled “Research on the Epidemiology of SARS-CoV-2 in Essential Response Personnel (RECOVER)” for estimating the incidences of asymptomatic and symptomatic infections and reinfections of SARS-CoV-2 in essential workers and for the rapid evaluation of COVID-19 vaccine effectiveness and immunogenicity against SARS-CoV-2 infection. In addition to these 2 primary objectives, data from the RECOVER study will be analyzed to assess a set of secondary objectives, including the risk factors for infection, the clinical spectrum of illness, the severity of infection after vaccination, and vaccine effectiveness against secondary infections within households.

## Methods

### Study Design

The RECOVER study has been designed to enroll and follow approximately 3000 essential workers for at least 18 months in 6 states. The primary objectives are to (1) estimate the incidences of asymptomatic and symptomatic SARS-CoV-2 infections and reinfections, and (2) evaluate COVID-19 vaccine effectiveness in preventing symptomatic and asymptomatic SARS-CoV-2 infections. Additional objectives include examining individual and occupational predictors of infection, and characterizing the clinical spectrum of COVID-19 illness. We also examine the association involving serum concentrations of per- and polyfluoroalkyl substances (PFAS), a component of some fire-suppression foams that has been associated with reduced immune response to vaccination [16]. Finally, we evaluate humoral and cellular immune responses to infection and vaccination, and estimate secondary infection rates within the households of vaccinated and unvaccinated RECOVER participants (Textbox 1).

The RECOVER study is funded by the US Centers for Disease Control and Prevention (CDC). The CDC and Abt Associates made key decisions on the study design, with scientific and operational input from investigators at each of the 6 study sites. Marshfield Clinical Research Institute in Marshfield, Wisconsin advised on laboratory methods for respiratory specimens in the study, and the CDC advised on the laboratory methods for serologic specimens. All study sites use a common protocol and data collection instruments approved by their institutional review boards (IRBs), as well as standard operating procedures.

**Objectives of the RECOVER (Research on the Epidemiology of SARS-CoV-2 in Essential Response Personnel) study.**

**Primary objectives**

- Determine the frequency of SARS-CoV-2 infection and COVID-19 illness among essential workers using both molecular and serologic diagnostic tests.
- Identify and describe SARS-CoV-2 reinfections among essential workers with prior SARS-CoV-2 infection as confirmed by reverse transcription polymerase chain reaction and/or SARS-CoV-2 serum antibody detection.
- Assess the effectiveness of SARS-CoV-2 vaccines in preventing SARS-CoV-2 infection and COVID-19 illness in essential workers. Examine vaccine effectiveness for different vaccine exposures, including different vaccine types and full versus partial adherence to recommended vaccine doses and timing.

**Secondary objectives**

- Assess the incidence of primary symptomatic and asymptomatic laboratory-confirmed SARS-CoV-2 infections in essential workers by examining observed frequencies within the context of characterized source populations.
- Examine the individual, occupational, and environmental predictors of SARS-CoV-2 infection and of asymptomatic infection versus symptomatic COVID-19 illness in essential workers.
- Describe the clinical characteristics and outcomes associated with COVID-19 in essential workers.
- Characterize the duration and severity of illness and examine the sociodemographic and health characteristics associated with prolonged or severe illness in essential workers.
- Determine the impact of COVID-19 on the indicators of functioning, including missed work, ability to complete normal work and home activities, and working while ill in essential workers.
- Determine the proportion of COVID-19 illnesses that are medically attended and examine the factors associated with seeking medical care and treatment in essential workers.
- Compare illness characteristics and duration among essential workers with primary infection versus reinfection with SARS-CoV-2.
- Evaluate the kinetics of immune responses to SARS-CoV-2 infection by comparing immune indicators from sera collected before or during illness with those collected after illness in essential workers.
- Examine antibody correlates of protection against SARS-CoV-2 reinfection in essential workers.
- Examine the duration of viral RNA detection associated with symptomatic COVID-19 illness in essential workers.
- Examine the interindividual variability in the magnitude and duration of viral RNA detection in essential workers.
- Assess the infectiousness of prolonged virus shedding in essential workers.
- Identify essential workers’ familiarity with personal protective equipment and other infection control measures or facility policies related to SARS-CoV-2, COVID-19, and pandemic response.
- Compare molecular diagnosis relying on different respiratory specimen types (eg, anterior nasal swabs versus saliva).
- Examine the association between serum concentrations of per- and polyfluoroalkyl substances (PFAS) in essential workers and the manifestations of COVID-19 illness and immune response to SARS-CoV-2 infection, including risk of reinfection and COVID-19 vaccine effectiveness.

**Additional pandemic vaccine objectives**

- Assist in the evaluation of the immunogenicity of pandemic SARS-CoV-2 vaccines by collecting sera from essential workers before and after vaccination and performing serologic and cellular immune response testing.
- Examine whether vaccine effectiveness is modified by sociodemographic characteristics, occupation, health status, or other risk factors in essential workers.
- Examine whether vaccination modifies COVID-19 illness severity, duration, and infectiousness (or viral shedding) among essential workers with breakthrough infections.
- Characterize the knowledge, attitudes, and practices related to new COVID-19 vaccines and examine the associations of knowledge, attitudes, and practices with subsequent vaccination behaviors (including vaccine refusal, hesitancy, or incomplete adherence to vaccination recommendations) among essential workers.
- Determine the association of serum PFAS concentration with SARS-CoV-2 infection, COVID-19 illness, and SARS-CoV-2 antibodies in essential workers.

**Exploratory objectives**

- Examine the kinetics of the immune response to SARS-CoV-2 infection through serial sampling of sera in essential workers.
- Examine the kinetics of viral shedding associated with SARS-CoV-2 infection and COVID-19 illness in essential workers.
- Examine individual heterogeneity in the use and host response to COVID-19 therapeutics and vaccines in essential workers.
- Examine cell-mediated immune responses (B cells, and CD4 and CD8 T cells) to SARS-CoV-2 infection in essential workers.
- Estimate the secondary attack rate of SARS-CoV-2 infection within households of essential workers.
- Estimate the effectiveness of available vaccines or antiviral prophylaxes and treatments to prevent secondary transmission within the households of essential workers as they become available.

### Setting

The RECOVER study consists of 6 health care system–based and academic research and hospital-based partner institutions with experience recruiting HCP and first responder cohort populations. Most of these study sites also serve as the usual source of medical care to a large proportion of the local community population, including essential workers, and are uniquely positioned to rapidly identify and recruit participants. The RECOVER study network includes the University of Arizona in Tucson, Arizona; Baylor Scott and White Health in Temple, Texas; Kaiser Permanente Northwest in Portland, Oregon; the University of Miami in Miami, Florida; St. Luke’s Hospital in Duluth, Minnesota; and the University of Utah in Salt Lake City, Utah (Table 1).

**Table 1:** RECOVER (Research on the Epidemiology of SARS-CoV-2 in Essential Response Personnel) study sites and site characteristics.

Variable	University of Arizona	Baylor Scott & White Health	Kaiser Permanente Northwest	University of Miami	St. Luke’s Hospital	University of Utah
Geographic area of the site	Tucson, Arizona	Temple, Texas	Portland, Oregon	Miami, Florida	Duluth, Minnesota	Salt Lake City, Utah
Participant catchment area	Pima County, Arizona	Bell County, Texas and cities of Temple, Belton, and Killeen, Texas	Northwest Oregon from Eugene, Oregon to Longview, Washington	Miami-Dade, Broward and Palm Beach Counties, Florida	Within 100 miles of Duluth, including northwest Wisconsin	Within 60 miles of Salt Lake City, Utah
Study population composition goal	14% HCP^a^ 37% first responders 49% EFWs^b^	70% HCP 10% first responders 20% EFWs	68% HCP 11% first responders 21% EFWs	9% HCP 64% first responders 27% EFWs	68% HCP 11% first responders 21% EFWs	45% HCP 29% first responders 26% EFWs
Communication of reverse transcription polymerase chain reaction results to participants	Study staff call with positive results; positive and negative test results sent by mail	Study staff call with positive results; positive and negative results added to participant’s EMR^c^ as a patient message	Study staff call with positive results; positive and negative test results sent by mail	Study staff call with positive results; positive and negative test results sent by email	For HCP, occupational health reports to participants. For first responders and EFWs, staff call with positive test results	Positive and negative test results emailed to participants
Communication of reverse transcription polymerase chain reaction results to the state or local health department	Positive and negative test results submitted to Arizona Department of Health Services	Positive test results submitted to Bell County Health Department	Positive and negative test results submitted to Oregon Health Authority and Washington Department of Health	Positive and negative test results submitted to Florida Department of Health	Positive test results submitted to Minnesota Department of Health or Wisconsin Department of Health	Positive and negative test results submitted to Utah Department of Health
Primary recruitment method for HCP and first responders	Recruiting from an existing Firefighter Cancer Cohort study and the University of Arizona Antibody Testing Initiative, as well as via fire station, police department, hospital, and clinic site visits	Recruiting from volunteers of previous research studies and employees within the Baylor health system via hospital and clinic site visits, targeted emails, and phone calls	Recruiting from volunteers of previous research studies and employees with Kaiser medical coverage via targeted emails and phone calls	Recruiting from an existing Firefighter Cancer Cohort study, as well as via fire station, police department, hospital, and clinic site visits	Recruiting from employees within the St Luke’s health system and surrounding area first responder companies via targeted emails to team leaders, emails to employee lists, and phone calls	Recruiting from employees within the University of Utah health system and surrounding area first responder companies via targeted letters to team leaders, emails to employee lists, and phone calls
Primary recruitment method for EFWs	Recruiting from employer groups via site visits, employer contacts, and the Arizona Antibody Testing Initiative	Recruiting from various local worker groups associated with existing university partners via emails and local radio advertisements	Recruiting from the population with Kaiser medical coverage via targeted emails and phone calls	Recruiting from various local worker groups associated with existing university partners via in-person outreach and email invitations	Recruiting from various local worker groups via social media, calls, emails, local radio advertisements, and current participant referrals	Recruiting from various local worker groups associated with existing university partners via social media, emails, and local radio/media advertisements

^a^HCP: health care personnel.

^b^EFW: essential and frontline worker.

^c^EMR: electronic medical record.

### Eligibility Criteria

Eligible essential workers include HCP, first responders, and EFWs who meet the following criteria: age at least 18 years; current work in a health care, first response, or another essential or frontline occupation; work for at least 20 hours a week; direct contact with people, defined as being within 3 feet of other people as part of job responsibilities; willingness to receive and respond to SMS text messages; and willingness to provide medical history via electronic medical record (EMR) access or health-related questionnaires. This study’s definition of “direct contact” (within 3 feet) does not require physical contact, but it is physically closer than that in the definition used to describe any contact for SARS-CoV-2 exposure (within 6 feet) to reflect high exposure conditions common to essential responder occupations [17]. Potential participants are excluded if they work less than an average of 20 hours per week, have already received a COVID-19 vaccine, or have participated in a COVID-19 prevention or treatment investigational trial in the 3 months prior to screening for the study.

### Recruitment

To minimize potential biases and ensure a diverse population of essential workers, each study site aims to enroll 500 to 700 participants using a 3-phase stratified recruitment approach according to sex, 2 age groups (18-39 years and 40+ years), and 4 occupational categories (primary HCP, support HCP, first responders, and EFWs). Primary HCP are physicians (Doctor of Medicine or Doctor of Osteopathic Medicine), physician assistants, dentists, and nurse practitioners, since these are typically underrepresented occupations in cohort studies of HCP [18]. Support HCP are all other personnel not included in the primary category. Each study site aims to recruit a minimum of 20 participants per occupation, age, and sex stratum. While sites are not required to incorporate racial and ethnic diversity directly into recruitment strata, targeted outreach for recruitment in diverse communities is conducted.

To gather the most information about protective immunity and reinfection over time, phase one recruitment includes HCP and first responders with current or any prior reverse transcription polymerase chain reaction (RT-PCR)–confirmed SARS-CoV-2 infection; HCP and first responders with an available baseline sera sample collected as a part of other research efforts; and HCP and first responders in difficult-to-fill recruitment strata, defined as particular ages or sex strata that are underrepresented at certain study sites (eg, female first responders). Phase two recruitment includes all other HCP and first responders to reach at least 400 participants per study site. Phase three recruitment includes EFWs, with a minimum of 100 enrollees per site. Recruitment and enrollment at each study site began in early August 2020. Site-specific methods for recruitment are provided in Table 1.

### Data Collection

**Figure 1:**
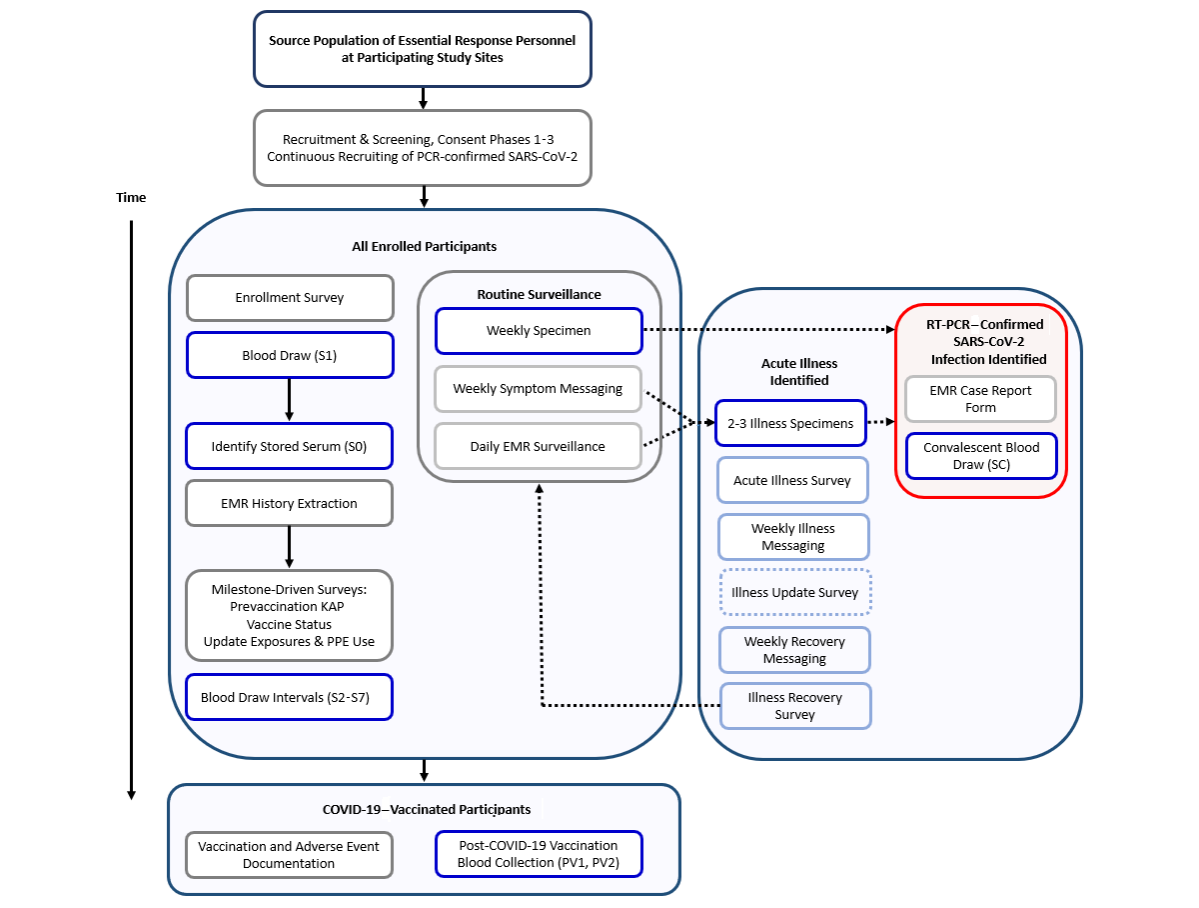
Research on the Epidemiology of SARS-CoV-2 in Essential Response Personnel (RECOVER) study activities. EMR: electronic medical record; KAP: knowledge, attitudes, and practices; PCR: polymerase chain reaction; PPE: personal protective equipment.

Data collection and site-level data management are conducted using Research Electronic Data Capture (REDCap), a browser-based metadata-driven software system (Vanderbilt University) [19]. Data collection occurs through SMS text messaging on the Twilio platform that integrates directly with REDCap, internet-based surveys, or telephone calls from study staff [20]. An overview of the study methods and data collection instrument delivery is provided in Figure 1.

### Enrollment Data

Following informed consent, participants are asked to complete an online survey to assess sociodemographic characteristics, occupation and work responsibilities, health status and behaviors, self-reported medical history, and use of PPE (Table 2). To further establish exposure levels, the health care setting and age of patients attended are collected, and both HCP and first responders are further asked about performing aerosol-generating procedures. All participants are asked to estimate exposures to persons, including patients, coworkers, and the public at work or in any setting, and to potential SARS-CoV-2 infection, as well as their PPE utilization.

**Table 2:** RECOVER (Research on the Epidemiology of SARS-CoV-2 in Essential Response Personnel) study data collection activities.

Variable	Screening	Enrollment	Weekly active surveillance	Follow-up survey 1	Follow-up survey 2	Acute illness surveys	Electronic medical records^a^
Essential worker status	Yes	No	No	No	No	No	No
Sociodemographics	Yes	Yes	No	No	No	No	Yes
Household size and age distribution	No	Yes	No	No	No	No	No
Occupation	Yes	Yes	No	Yes	Yes	No	No
Location of work	No	Yes	No	No	No	No	No
Patient care responsibilities (HCP^b^ only)	No	Yes	No	No	No	No	No
Typical hours worked	No	Yes	No	No	No	No	No
Health status and risk behaviors	No	Yes	No	Yes	Yes	No	No
Recent care for acute respiratory illness	No	Yes	No	Yes	Yes	No	Yes
Chronic medical conditions and pregnancy	No	Yes	No	Yes	Yes	No	Yes
Assessment for current CLI^c^	No	No	Yes	No	No	No	No
SARS-CoV-2 testing history	Yes	No	Yes	No	No	No	Yes
COVID-19 diagnosis history	Yes	No	No	No	No	No	Yes
Medical care for COVID-19	Yes	No	No	No	No	No	Yes
Contact with confirmed or suspected COVID-19	No	Yes	Yes	Yes	Yes	No	No
Contact with patients, customers, or the public	No	Yes	Yes	Yes	Yes	No	No
PPE^d^ utilization	No	Yes	Yes	Yes	Yes	Yes	No
PPE concerns	No	Yes	No	No	No	No	No
Knowledge, attitudes, and practices related to new COVID-19 vaccines	No	No	No	Yes	Yes	No	No
COVID-19 vaccination status	No	No	No	Yes	Yes	No	Yes
Influenza vaccination status	No	No	No	Yes	Yes	No	No
Employee records of illness absence	No	No	No	No	No	No	Yes

^a^Electronic medical records are available at the Baylor Scott & White Health, Kaiser Permanente Northwest, St. Luke’s Hospital, and University of Utah sites.

^b^HCP: health care personnel.

^c^CLI: COVID-19–like illness.

^d^PPE: personal protective equipment.

### Follow-Up Surveys

Approximately every 3 months after enrollment, participants are sent a follow-up survey to allow them to update information on their health status, job characteristics, potential exposures to COVID-19, and use of PPE. These surveys also assess participant knowledge, attitudes, and practices regarding COVID-19 vaccines; participant intention to be vaccinated; and, after each vaccination dose, any adverse reactions. Influenza vaccination status is assessed separately by self-report (including type of vaccine, lot number, and date of vaccination) starting approximately 2 months after the seasonal influenza vaccines become available each year.

### Active Surveillance

Study participants are followed weekly for the duration of the study period with active surveillance for SARS-CoV-2 infection and symptomatic COVID-19–like illness (CLI). Participants are contacted by SMS text message to determine the presence of one or more of the following symptoms of CLI in the past 7 days: fever, cough, shortness of breath, sore throat, diarrhea, muscle or body aches, and change in smell/taste. In October 2020, the case definition was modified to include chills based on an evolving understanding of the symptoms associated with COVID-19 [21-23]. These questions serve as a baseline for when participants are subsequently asked exposure and PPE utilization questions through the weekly active surveillance SMS text message, such that data are collected monthly.

Participants who report no CLI are asked 1 of the following 4 rotating questions about contacts and sleep in the past 7 days: (1) hours spent in direct contact with persons with suspected or confirmed SARS-CoV-2 at work, (2) hours spent in direct contact with other people at work, (3) hours spent in direct contact with people outside of work, and (4) quality of sleep. Each question about contact is followed by asking about the percentage of time they wore PPE. Participants are then reminded to collect their weekly respiratory specimens and ship them to Marshfield Clinical Research Institute for testing.

Participants who report experiencing CLI are asked to self-collect the standard respiratory specimen, an additional anterior nasal dry foam swab, and a saliva sample. The participant is sent an acute illness survey, including questions regarding symptoms since onset, self-rated health today, ability to perform normal activities, and medical utilization. The survey also asks about direct contact within the past 7 days with known or suspected COVID-19 cases at work and outside work, direct contact with other people, and PPE use during those contacts. The weekly SMS text messages are modified to assess if their illness is continuing and to gauge their ability to conduct normal activities. Participants who report continuous symptoms ≥7 days after illness onset are asked to complete an illness update survey to assess new symptoms, illness severity, and medical utilization.

Once participants report that they are no longer experiencing illness symptoms, they are asked to complete an illness resolution survey to assess symptoms, severity of illness, and medical utilization over the course of the illness, and to estimate their current recovery progress (defined as feeling 0%-100% of “normal”). Until a participant reports ≥90% recovery progress, the weekly surveillance messaging will continue to ask about recovery progress. Once a participant reports ≥90% recovery, the weekly messaging will return to the standard question set.

Acute illness may also be reported any time during the week to study staff. At study sites with EMR capabilities, acute illness may also be identified through daily monitoring of medical visits for an acute respiratory illness.

### EMRs

When available, data from EMRs are abstracted and extracted to count medical visits for acute COVID-19 illness, identify influenza and COVID-19 vaccination data, and assess chronic medical conditions for the 12 months prior to enrollment and through the end of the study. The EMR extraction uses International Statistical Classification of Diseases and Related Health Problems, 10th revision (ICD-10) codes for all ambulatory medical encounters and all hospital admissions. Chronic medical conditions and vaccination status are assessed by self-report for participants with or without access to EMR data.

### COVID-19 Vaccination Data

COVID-19 vaccination status is assessed through multiple methods to ensure complete and near real-time data capture, which is a critical component for scheduling postvaccination blood collection and monitoring for receipt of additional doses. Participants are asked in advance to proactively self-report if they are scheduled for COVID-19 vaccination or to contact local study staff as soon as possible after receipt of each vaccine dose. Throughout the study, participants receive brief periodic surveys via SMS text message links to report vaccination. Additionally, sites query participants’ EMR and their state immunization information system to obtain COVID-19 vaccination status or history. If COVID-19 vaccination is self-reported, participants are asked to send a digital photograph of the vaccination card provided at vaccination using secure file transfer. Study staff cross-check vaccination information received from multiple sources to ensure accuracy.

### Household Transmission Data Collection

To assess the transmissibility of SARS-CoV-2 in vaccinated and unvaccinated individuals, all members of a RECOVER participant’s household (adults and children) are asked to join the study for a 10-day period if an RT-PCR–confirmed SARS-CoV-2 infection is identified in the RECOVER participant. These daily household study activities began in February 2021. Once a SARS-CoV-2 infection is confirmed, participants with at least one other household member are contacted by study staff to enroll both the RECOVER participant and their household members into this component of the study. Data collection activities include a brief enrollment interview to ascertain demographics, vaccination status, and prior SARS-CoV-2 infection among household members. All enrolled household members are asked to self-collect a mid-turbinate nasal swab each day for 10 days, regardless of infection status. On a daily collection form, participants are asked to indicate the day their specimen was collected and the presence or absence of CLI symptoms.

### Retention and Adherence

Study sites implement several strategies to increase retention among their enrolled participants. These include robust incentive programs that vary by study site within the confines of local IRB requirements, monthly newsletters with aggregated study data and results, and opportunities for participant feedback on study activities. When possible, study sites also combine study activities within a single contact or visit to reduce participant burden and increase retention. For example, a visit for a blood draw may also include catching up on any missed survey instruments and collecting the weekly respiratory specimen so the participant does not have to personally ship the specimen that week.

Study sites regularly monitor participant adherence to main study activities, including through automated reports on missing weekly surveillance swabs, automated survey reminders, phone calls to the participants to collect missing information, and mailed letters detailing missed activities. Upon consecutive poor adherence to study activities, study staff reach out to the participants to identify possible solutions, such as changing their specimen shipping procedures, adjusting the day or time of their weekly surveillance survey, or altering the location for their blood collection appointments. If adherence remains low after these modifications or there is general nonresponse from the participants, they will be considered for withdrawal from the study.

### Laboratory Methods: Respiratory Specimens

Each week, participants collect a mid-turbinate nasal specimen using a flocked swab placed in viral transport medium (VTM). Upon the onset of CLI symptoms, participants self-collect a mid-turbinate flocked nasal swab in VTM, as well as a dry foam anterior nasal swab and saliva. Participants are provided with training and materials for the self-collection of each specimen type at enrollment. When collecting saliva, participants are instructed to avoid eating, using tobacco products, or brushing their teeth for 30 minutes prior to collection. They then deposit approximately 2 mL of saliva into a collection tube with a funnel. Participants are given written and visual detailed instructions for collecting, storing, and submitting specimens by dropping them off at a specified location, having them picked up by an at-home courier, or shipping them according to guidance and specifications from the US Food and Drug Administration.

All respiratory specimens are shipped on the day of collection unfrozen (on ice packs) to Marshfield Clinical Research Institute for testing. On a daily basis, the Marshfield Clinical Research Institute performs a CDC-specified RT-PCR assay to ascertain infection with SARS-CoV-2. Study staff at all sites inform participants of their test results when they are positive for SARS-CoV-2, noting that results are not meant to replace recommended clinical tests. Some study sites also notify participants of negative test results. Each study site complies with the reporting requirements of their state and local public health departments. Remaining aliquots of all study specimens are sent to a CDC-designated facility for additional virus characterization (including but not limited to genetic sequencing and novel severity markers), banking, and storage.

### Laboratory Methods: Serum and Blood Specimens

All participants contribute 20 mL of whole blood at least 4 times per year, including at enrollment and approximately every 3 months thereafter. Participants who experience an immune-modifying event, including SARS-CoV-2 infection indicated by a confirmed positive RT-PCR test or vaccination, have blood drawn approximately 28 days after infection and 14 to 28 days after each vaccine dose. The next blood collection occurs 3 months after any event-related blood draw to keep the burden on the participant to a minimum. All participant blood collections occur in-person at the study sites and are performed by trained study staff or affiliated phlebotomists.

Whole blood is collected and processed by the study site laboratory using CDC guidelines for serum collection. The serum specimen is divided into aliquots. All specimens are stored in a −20℃ or colder freezer and shipped to the central study CDC laboratory for SARS-CoV-2–specific antibody detection and characterization. A serum aliquot from the first or second serum collection is stored for analysis of PFAS. Additional specimens from later in the study will be kept for potential future analysis of longitudinal changes in PFAS concentrations.

### Ethical Approval and Ethical Considerations

The study protocol and procedures were reviewed and approved by the following 5 IRBs: Baylor Scott and White Research Institute IRB; Kaiser Permanente Northwest IRB (IRB of record for the CDC and Abt Associates); St. Luke’s Hospital Duluth IRB; University of Miami Human Subjects Research Office (IRB of record for University of Arizona); and University of Utah IRB. All participants have completed informed consent. Small gifts or stipends are given to participants at study milestones on a site-specific basis. Each study site follows local policies for employer notification of positive SARS-CoV-2 test results and reporting to state or local health departments.

### Sample Size

The RECOVER study has enrolled approximately 500 participants at each of the 6 sites for a total of 3000 participants. The required sample size to achieve incidence and vaccine effectiveness objectives accounts for the expected cumulative incidence of symptomatic and asymptomatic SARS-CoV-2 infections in the essential worker population, vaccine effectiveness, and study attrition. Early reports of the SARS-CoV-2 incidence and seroprevalence in essential workers ranged from 4% to 18%; thus, we assume a 10% cumulative incidence of infection among those with no documented RT-PCR infection before enrollment and 10% attrition [24-26]. We expect the cumulative incidence of reinfection to be 1% among those with a documented RT-PCR–positive SARS-CoV-2 infection before enrollment. Using these assumptions, we have determined that the sample size would have at least 80% power to detect a true percentage of 4% symptomatic infection and 8% asymptomatic infection.

Vaccines for COVID-19 were not available at the start of the study, but started to become available at each site in mid-December 2020. To account for vaccination status as a time-varying exposure, we used a Monte Carlo simulation to estimate the statistical power to detect a vaccine effectiveness of interest. Given the vaccine coverage, SARS-CoV-2 incidence, and underlying effect size, expected person-time of participants while unvaccinated and vaccinated and time to infection events were generated from the equation proposed by Austin, and a frailty/marginal model was fitted to estimate vaccine effectiveness [27]. We considered vaccine uptake to be 50% to 80% by occupation, accounting for the highest uptake and prioritization for HCP and first responders, and the lowest uptake and prioritization for EFWs. Assuming a monthly attack rate of 1% to 2% and a conservative vaccine effectiveness of 60% to 75%, sufficient statistical power will be reached with 6 months of participant time contributed.

### Data Analysis

The primary outcome of SARS-CoV-2 infection incidence will be estimated overall and according to asymptomatic and symptomatic presentation using individual participant contributions of person-time from the time of study entry. To control for the contribution of age, sex, and occupation to the risk of infection, incidence will be estimated using negative binomial models with a person-time offset. The association of individual, occupational, and environmental predictors with SARS-CoV-2 infection will be assessed using a mixed-effects Cox proportional hazards model, incorporating study site as a random effect.

Vaccine effectiveness against SARS-CoV-2 infection and COVID-19 illness will be calculated using the Andersen-Gill extension of the Cox proportional hazards model, which allows vaccination status to be time varying. Unadjusted vaccine effectiveness is calculated as 100% × (1−hazard ratio). The effect of the following covariates on vaccine effectiveness estimates will be considered for all outcomes: study site, sociodemographic characteristics, occupation, self-reported occupational SARS-CoV-2 exposure and PPE utilization, and underlying health status. We will calculate stabilized weights to create a pseudopopulation where measured covariates are independent of vaccination. In this way, we can properly measure the causal association between vaccine and infection. Depending on sample size, vaccine effectiveness may be calculated by age group, full versus partial vaccination, and vaccine type, if multiple products are in use.

### Cell-Mediated Immunity Substudy

The Baylor Scott and White Health and Kaiser Permanente Northwest sites are conducting a substudy to compare cell-mediated immune response to SARS-CoV-2 infection and vaccination, and metabolome and microbiome diversity among participants with symptomatic and asymptomatic SARS-CoV-2 infections or vaccination. To achieve these objectives, 3 subgroups are invited to participate, and are independently screened for eligibility, consented, and enrolled. These 3 subgroups include those with symptomatic or asymptomatic SARS-CoV-2 infection, COVID-19 vaccine recipients, and uninfected and unvaccinated controls. SARS-CoV-2–infected individuals are invited to participate upon diagnosis, and controls are time-matched with a 1:1 case-control ratio. Participants provide a self-collected dry mid-turbinate nasal swab for metagenomic characterization of the microbiome or virome, and whole blood for serum and peripheral blood mononuclear cell (PBMC) extraction. Serum and plasma aliquots will be used to supplement existing antibody testing time points with Ig-Seq to determine antibody clonotypes, metabolome, and VirScan multipathogen assays. The PBMCs will be used for human B- and T-cell repertoire analysis, phenotypic characterization of innate lymphoid cells with multiparametric flowcytometry, and single-cell transcriptomics. The substudy protocol and procedures have been reviewed and approved by the Baylor Scott and White Research Institute IRB and the Kaiser Permanente Northwest IRB (IRB of record for the CDC and Abt Associates).

### Serial Serology Substudy

The St. Luke’s Hospital site is conducting a substudy to determine time to seroconversion from a positive nasal swab and define the duration of seropositivity in those who seroconvert. All participants are invited to join the substudy, and those enrolled provide 7 mL of whole blood every 4 weeks, the serum of which is divided into 3 aliquots and frozen for serologic testing. The frequency of blood draws is increased to every 2 weeks for individuals with known SARS-CoV-2 infection by RT-PCR prior to enrollment in the RECOVER study or at any time after enrolling in the study. Serum aliquots will be tested for antibodies to SARS-CoV-2 to determine the kinetics of the immune response. The substudy protocol and procedures have been reviewed and approved by the St. Luke’s Hospital Duluth IRB and the Kaiser Permanente Northwest IRB (IRB of record for the CDC and Abt Associates).

## Results

The study observation period began in August 2020 and is expected to continue through spring 2022. There are 2623 actively enrolled RECOVER participants, including 280 participants who have tested positive for SARS-CoV-2 by RT-PCR through the study. Enrollment is ongoing at 3 of the 6 study sites (3 study sites have completed enrollment). In June 2021, the RECOVER network published estimates of vaccine effectiveness [28].

## Discussion

The RECOVER cohort continues to enroll participants in a unique research opportunity to estimate the incidence of asymptomatic and symptomatic SARS-CoV-2 infections in essential workers. It also provides early estimates of COVID-19 vaccine effectiveness against both infection and transmission of SARS-CoV-2 to household members of the RECOVER cohort. Here, we describe the recruitment, data gathering, active surveillance, laboratory testing, and planned data analysis procedures for this prospective longitudinal cohort study of essential workers representative of working-age adults in these professions. Through weekly collection and testing of respiratory specimens from all participants, regardless of the presence of CLI symptoms, we are able to examine the occurrence of asymptomatic and presymptomatic SARS-CoV-2 infection, which has been shown to represent the highest risk of transmission [29]. The longitudinal study design and collection of both periodic and weekly information about behaviors and risk factors will inform measurement of primary outcomes, including incidence and vaccine effectiveness estimations. Further, our study includes blood specimens from all participants, which affords the opportunity to identify infections that may not be captured by RT-PCR–based testing or that may have occurred before the participant joined the cohort. We are also able to assess the kinetics of the immune response, both postinfection and postvaccination.

In addition to the longitudinal design and weekly specimen testing, the RECOVER study has geographic variability and a stratified recruitment design. The study is recruiting participants from geographically diverse areas of the United States, which will allow us to evaluate trends in SARS-CoV-2 infection in areas with varying prevalence levels, policies around social distancing and large gatherings, and rates of COVID-19 vaccine uptake. The geographic diversity may also help to identify SARS-CoV-2 variants and help inform how vaccine effectiveness is affected by these variants. The study utilizes a stratified recruitment approach to ensure there is sufficient variability in sex, age, and occupational categories within the cohort. This approach intentionally creates an opportunity to examine risks associated with heterogeneity in sociodemographic characteristics and improves the ability to compare and collapse data across study sites if necessary. This systematic approach is also intended to minimize convenience sampling, which can introduce known and unknown biases.

Our study will face challenges and have limitations. First, ensuring consistent adherence to study procedures over a long period of time is challenging. To overcome this obstacle, site staff will strive to develop a relationship with participants and provide feedback regarding strategies the study may implement to help participants adhere to study methods. Second, while the diversity of the occupational categories will enhance the overall representativeness of the RECOVER study population, it may increase the complexity of specimen collection by including individuals who may not work near clinical facilities. To facilitate submission of respiratory specimens, participants may choose to ship their specimens overnight, drop them off at a study facility, or have them picked up from home by a medical courier. Third, we are relying on self-collected mid-turbinate nasal swabs, which have ranged in sensitivity from 75% to 90% in studies comparing SARS-CoV-2 detection in swabs collected by nonclinical subjects with that in swabs collected by HCP [30,31]. This limitation will be minimized by the volume of participants who are HCP and are familiar with swab collection; regardless, all participants are trained by study staff in proper sample collection [31]. Finally, unforeseen shipping interruptions may delay specimens and impact viral detection. Studies have demonstrated nasal swab specimen stability at ambient temperatures for up to 9 days, and specimens are shipped with ice packs as a precaution [32].

The RECOVER cohort is designed to answer critical questions regarding SARS-CoV-2 incidence and COVID-19 vaccine effectiveness among essential workers. Data collected through the cohort are expected to inform public health decisions related to PPE use, exposure risk factors, assessment of humoral and cellular responses to infection and vaccination, evaluation of COVID-19 vaccine effectiveness, and policies for returning to work after infection. Furthermore, the RECOVER cohort will have the capacity to examine variant strains of SARS-CoV-2 and their impacts on vaccine effectiveness.

## Abbreviations

CDC: US Centers for Disease Control and Prevention
CLI: COVID-19–like illness
EFW: essential and frontline worker
EMR: electronic medical record
HCP: health care personnel
IRB: institutional review board
PBMC: peripheral blood mononuclear cell
PFAS: per- and polyfluoroalkyl substances
PPE: personal protective equipment
RECOVER: Research on the Epidemiology of SARS-CoV-2 in Essential Response Personnel
REDCap: Research Electronic Data Capture
RT-PCR: reverse transcription polymerase chain reaction
VTM: viral transport medium
